# Structure of the mycobacterial ESX-5 type VII secretion system pore complex

**DOI:** 10.1126/sciadv.abg9923

**Published:** 2021-06-25

**Authors:** Katherine S. H. Beckham, Christina Ritter, Grzegorz Chojnowski, Daniel S. Ziemianowicz, Edukondalu Mullapudi, Mandy Rettel, Mikhail M. Savitski, Simon A. Mortensen, Jan Kosinski, Matthias Wilmanns

**Affiliations:** 1 European Molecular Biology Laboratory, Hamburg Unit, Notkestrasse 85, 22607 Hamburg, Germany.; 2 European Molecular Biology Laboratory, Heidelberg, Germany.; 3 Centre for Structural Systems Biology (CSSB), Hamburg, Germany.; 4 Structural and Computational Biology Unit, European Molecular Biology Laboratory, Meyerhofstrasse 1, 69117 Heidelberg, Germany.; 5 University Hamburg Clinical Centre Hamburg-Eppendorf, Martinistrasse 52, 20246 Hamburg, Germany.

## Abstract

The ESX-5 type VII secretion system is a membrane-spanning protein complex key to the virulence of mycobacterial pathogens. However, the overall architecture of the fully assembled translocation machinery and the composition of the central secretion pore have remained unknown. Here, we present the high-resolution structure of the 2.1-megadalton ESX-5 core complex. Our structure captured a dynamic, secretion-competent conformation of the pore within a well-defined transmembrane section, sandwiched between two flexible protein layers at the cytosolic entrance and the periplasmic exit. We propose that this flexibility endows the ESX-5 machinery with large conformational plasticity required to accommodate targeted protein secretion. Compared to known secretion systems, a highly dynamic state of the pore may represent a fundamental principle of bacterial secretion machineries.

## Teaser

The structure of the mycobacterial ESX-5 secretion system reveals a central transmembrane pore.

## INTRODUCTION

Mycobacterial pathogens cause more than one million deaths each year (*1*). Key to their pathogenicity is the secretion of a wide range of virulence proteins via type VII secretion systems (T7SSs) (*2*). The ESX-5 T7SS is found almost exclusively in slow growing, pathogenic mycobacteria (*3*) and plays a key role in nutrient uptake and immune modulation during an infection (*4*–*6*). The importance of ESX-5 and other ESX secretion systems (ESX-1 to ESX-4) for the virulence of mycobacterial pathogens makes these membrane-spanning machineries a key target for the development of novel therapeutics (*7*). However, despite its importance, we still lack high-resolution structural information of a fully assembled core complex, which is critical for a mechanistic understanding of ESX-5 function and for informing drug development.

A previous low-resolution model of the ESX-5 complex revealed the presence of a hexameric core complex with dimensions suggesting that it spans the inner mycobacterial membrane (*8*). Unexpectedly, recent high-resolution structures of the ESX-3 complex from *Mycobacterium smegmatis* revealed a dimeric complex, instead of the expected hexameric pore assembly, and led to the suggestion that the dimer represented a building block of the full assembly. However, because of incomplete density for the membrane helices of EccC_3_, an accurate model of a hexameric pore could not be generated (*9*, *10*). To address this question and reveal details of the ESX-5 structure, dynamics, and assembly, we used an integrated structural biology approach using single particle cryo–electron microscopy (cryo-EM), mass spectrometry (MS)–based cross-linking, and integrative modeling. Our data revealed a highly dynamic central ESX-5 pore embedded within a rigid membrane scaffold formed by six protomeric units, with a diameter ranging from 4 to 10 Å, suggesting that we captured ESX-5 in the partially open, secretion-competent state. Moreover, our structure showed an unanticipated break in symmetry between the membrane and cytoplasmic regions, with a sixfold symmetric arrangement, and the periplasmic region, which displayed a twofold symmetry. We propose that this conformational plasticity and the flexibility in both the periplasmic and cytoplasmic regions are critical for protein substrate recognition, transport, and release.

## RESULTS

### Overall structural organization of the hexameric ESX-5 pore complex

**Fig. 1:**
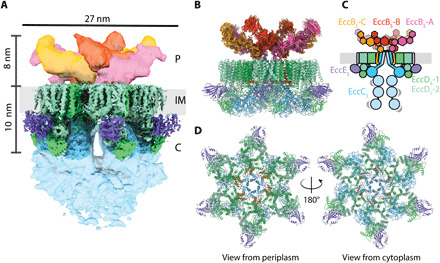
Overall architecture of the ESX-5 complex. (**A**) Composite EM map of the ESX-5 complex highlighting the periplasmic (P), inner-membrane (IM) and cytosolic (C) regions of the complex. Periplasmic regions are colored in yellow, orange, and pink. TM and membrane-proximal cytosolic region are colored according to the scheme shown in (C). The distal cytosolic segment tentatively corresponding to the ATPase domains is shown in light blue, and the mycobacterial inner membrane (IM) is indicated in light gray. (**B**) Side view of the composite model of the ESX-5 complex generated by combining the atomic coordinate model, with an ensemble of models built using integrative approaches. (**C**) Schematic of the ESX-5 complex showing the organization of the components EccB_5_ (orange), EccC_5_ (light blue), EccD_5_ (green, light green), and EccE_5_ (purple). The different EccB_5_ domains are colored to show their location in the segmented EM map in (A). Low-resolution density has been shown as a lighter shade for EccC_5_ and EccE_5_. (**D**) Top and bottom views of the rigid core of the ESX-5 membrane complex.

To elucidate the structure of the T7SS, we purified the *Mycobacterium xenopi* ESX-5 complex for single-particle cryo-EM analysis (fig. S1). The initial 3.9-Å resolution map calculated without imposed symmetry revealed substantial differences in the interpretability of different regions (fig. S2). Consistent with the previous low-resolution model (*8*), the membrane and cytoplasmic regions of the complex both displayed sixfold symmetry, with the latter showing a larger extent of positional disorder toward the periphery of the complex. We therefore generated two new maps imposing C6 symmetry for modeling these regions: a global map with a 3.4-Å resolution and a locally refined map of the cytosolic part of the protomeric units at 3.0-Å resolution (Fig. 1A and table S1). This enabled the de novo building of the transmembrane (TM) and membrane-adjacent cytoplasmic domains to 88% completeness with a detectable sequence register (EccB_5_ 18-73; EccC_5_ 12-417; EccD_5_-1 23-502; EccD_5_-2 18-494; and EccE_5_ 95-332) (Fig. 1D and fig. S4).

**Fig. 2:**
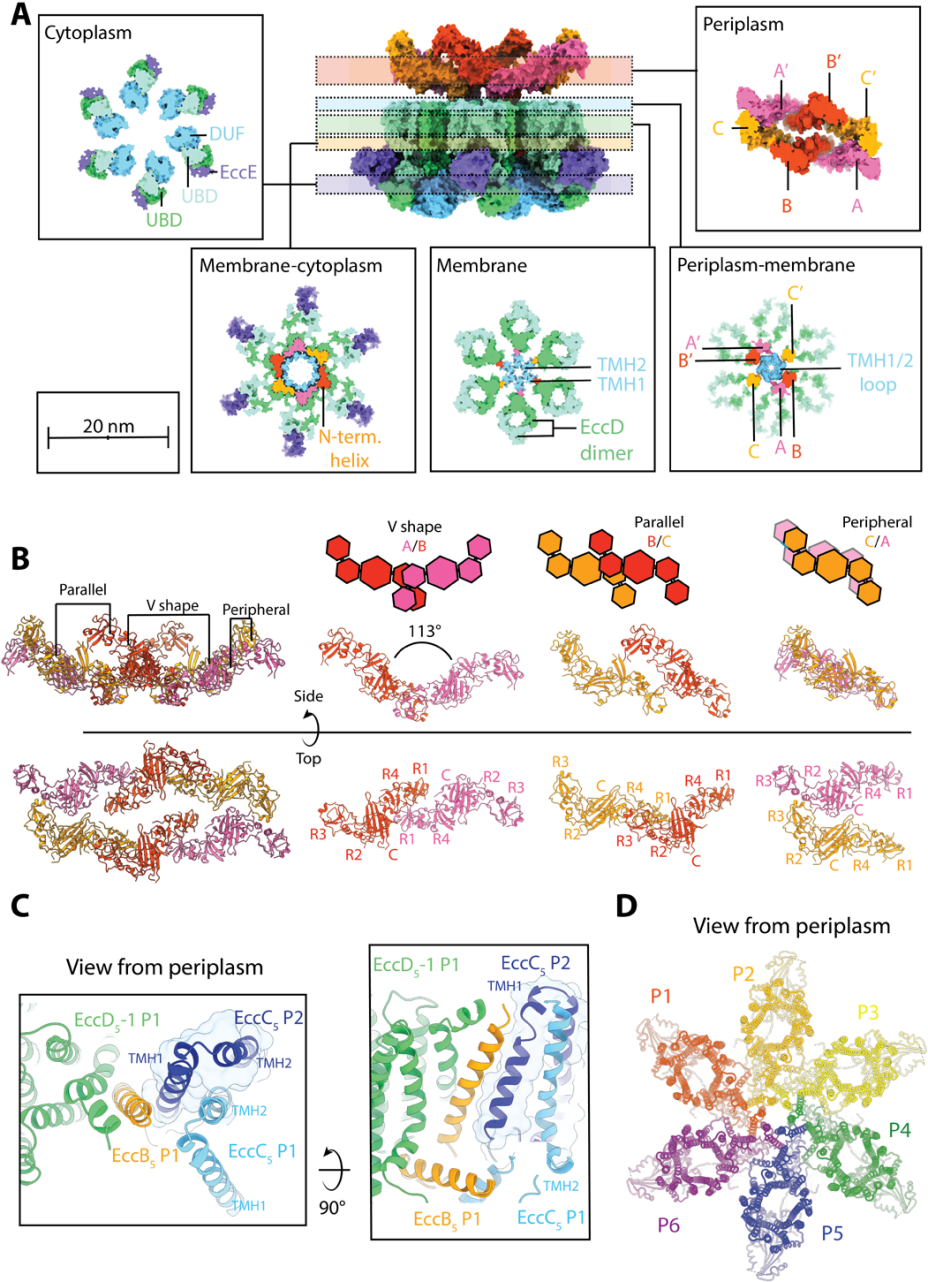
Subunit interactions and assemblies within the ESX-5 complex. (**A**) Cross-sections of the ESX-5 complex displayed as a surface representation of the atomic model shown as insets. Each inset highlights the subunit/subunit interactions occurring on the periplasmic, periplasmic-membrane, membrane, membrane-cytoplasmic, and cytoplasmic level. Key features described in the text are highlighted. (**B**) Overview of the assembly of EccB_5_ in the periplasm into defined dimers, parallel, V-shape, and peripheral. In the parallel dimer, EccB_5_-B forms an interface with EccB_5_-C, where B and C are shifted with respect to each other by about 5 nm and is stabilized by interactions between the central C-domains: (EccB_5_-B)/R1 + R4 (EccB_5_-C) and C (EccB_5_-C)/R2 + R3 (EccB_5_-B). The trimers along the long axis of the keel-shaped arrangement form the peripheral dimeric interface, mediated by R2 + R3 of EccB_5_-C from one trimer and by R2 + R3 of EccB_5_-A′ from the other trimer. These three EccB_5_ dimers assemble into the characteristic dimer-of-trimer arrangement of the periplasmic face structure of the overall ESX-5 complex (left), which does not follow the sixfold symmetry of other parts of the complex. In the top view, the subdomains in each EccB_5_ protomer are labeled, indicating specific EccB_5_ domain/domain interactions for the three distinct EccB_5_ dimeric interfaces observed. (**C**) Interprotomer interactions occurring between the EccC_5_ TMH1 arising from protomer 1 (P1) and EccB_5_ TMD arising from protomer 2 (P2). The two panels represent the top and side view, respectively. (**D**) Overview of domain swap interactions occurring between protomeric units when viewed from the periplasm. Each protomer has a different color.

To model the periplasmic region of the ESX-5 complex, we generated a third map without imposed symmetry restraints with a resolution of 4.6 Å. In contrast to the sixfold symmetry of the membrane and cytoplasmic regions, the periplasmic region of the ESX-5 secretion complex displayed an approximate C2 symmetry. Since the resolution of this map was not sufficient for de novo model building, we used a homology model of the periplasmic EccB_5_ domain (*11*) and distance restraints obtained from cross-linking MS to build an integrative ensemble model. The resulting model was composed of all six copies of EccB_5_ (Figs. 1, B and C and 2B) at an estimated precision of 7 Å (Materials and Methods, fig. S5, and table S2). Taking the cytosolic, TM, and periplasmic segment models together, we generated a model of the overall architecture of the complete ESX-5 pore complex (Fig. 1).

### Architecture of the ESX-5 protomer

Because of the observed sixfold symmetry of the TM and cytosolic segments of the ESX-5 complex, we first defined its overall arrangement as consisting of six identical protomeric subcomplexes. Each of these protomers comprises EccB_5_, EccC_5_, two copies of EccD_5_, and EccE_5_ (Fig. 2A and fig. S4). Within each protomeric unit, EccD_5_ was observed to assume an elliptical ring-shaped dimer composed of 22 (11 per EccD_5_ monomer) TM helices (TMHs) forming the core of the protomer. We observed that one of the EccD_5_ molecules is proximal and the second one is distal to the central pore, and we annotated them as EccD_5_-1 and EccD_5_-2, respectively. The EccD_5_ dimer was stabilized by hydrophobic interactions between TMH9 and TMH10 from EccD_5_-1 and TMH1 and TMH2 from EccD_5_-2 (Fig. 2, membrane inset, and fig. S3D).

The asymmetric ring-shape arrangement of EccD_5_-1 and EccD_5_-2 pairs were found to enclose a cavity in the membrane that is partially filled with density, which we attributed to copurified lipids that were detected in our liquid chromatography–MS (LC-MS) analysis of the purified complex (fig. S3, D and E and table S3). We noted that the EccD_5_ ring-like arrangement is further supported by interactions at the cytoplasmic face formed by TMH6 and the elongated TMH7, connected by an extended loop. Because of the approximate twofold symmetry of each EccD_5_ dimer, TMH11 of EccD_5_-2 was found most distal, and TMH11 of EccD_5_-1 is the closest to the central pore. Both TMH11 helices displayed a distinct diagonal orientation with respect to the remaining EccD TMHs and the pore axis (see below). In our model, contacts between TMH11 from EccD_5_-1 and the only EccB_5_ TMH establish a scaffold for the central pore. Although we observed that EccD_5_-2 TMH11 interacts with the TM domain of EccE_5_ in a similar manner, we have not included the TMHs of EccE_5_ in our high-resolution model due to weaker density, indicative of flexibility at the periphery (fig. S4).

In addition, we noted that EccD_5_ also acts as a main connector for the cytoplasmic regions of the ESX-5 protomer (Fig. 2A, cytoplasm inset, and fig. S4, A to D). The ubiquitin-like (Ubl) domains of EccD_5_ dimerize and interact with the cytoplasmic domain of EccE_5_ at the periphery of the ESX-5 complex. On the cytosolic side of the central pore, the EccD_5_ Ubl domain formed interactions with the EccC_5_ domain of unknown function (DUF), which follows the EccC_5_ stalk domain. The stalk domain is flanked by the long loop between TMH6 and TMH7 of EccD_5_-1 as well as the linker between EccD_5_-2 TMH1 and the EccD_5_-2 Ubl domain (fig. S4, B and D). Besides these interactions at the interface between the membrane and the cytoplasm (Fig. 2A, membrane cytoplasm inset), we observed several specific electrostatic interactions of the EccC_5_ stalk domain with the N-terminal EccB_5_ helix, situated parallel to the membrane. The density of our map was of sufficient quality to identify some of the electrostatic interactions unambiguously ( fig. S4F). As for the membrane region, the EccD_5_ Ubl domain dimer seems to act as a central scaffold for the other ESX-5 cytosolic subunits (fig. S4D).

In our structure, the DUF domain was the most distal rigid component at the cytosolic face of the ESX-5 complex. We attributed the less interpretable density for the remaining C-terminal part of EccC_5_ to the increased flexibility of adenosine triphosphatase (ATPase) domains 1 to 3, as previously observed (*8*–*10*). To examine the conformational space of ATPase domains further, we generated the C1 map at low threshold values (fig. S6A). Integrative modeling using the EM map and cross-linking distance restraints showed that six copies of the EccC_5_ C terminus fit into the density in multiple conformations, ranging from a cylindrical closed arrangement to assemblies with increasing degrees of opening (fig. S6A). The flexibility of EccC appears to be a common feature of the ESX systems (*9*, *10*), suggesting that this flexibility is key to the function of the T7SS.

### EccB_5_ forms an elongated interaction network around a central cleft in the periplasm

The EccB_5_ model at the periplasmic face showed two distinct EccB_5_ trimers arranged with an approximate twofold symmetry (EccB_5_-A/EccB_5_-B/EccB_5_-C used for further characterization below and EccB_5_-A′/EccB_5_-B′/EccB_5_-C′, highlighted in Fig. 2A, periplasm inset). The two trimers formed an elongated keel-shaped assembly with overall dimensions of 20 nm in length, 10 nm in width, and 8 nm in height divided by a central cleft. The assembly was formed by dimers of trimers with three distinct EccB_5_ dimeric arrangements, denoted as “V-shape,” “parallel,” and “peripheral” (Fig. 2B). Of note, in our ESX-5 structure, the angle between the two V-branches in each of the two EccB_5_ trimers was 113° (mean value; fig. S7), which is substantially different from the 85° angle of V-shaped EccB_3_ conformation found in the ESX-3 dimer structure (*9*). However, the angles between the principal axis of the periplasmic domains of the protomers within each of the two EccB_5_ trimers and the axis of the respective TM helix vary substantially (fig. S7A), demonstrating substantial flexibility of the hinge between the TM helix and the periplasmic domain within each EccB_5_ subunit. Our data thus indicate that the transition of hexameric symmetry observed for the TM section of the ESX-5 pore complex and lower symmetry of the elongated periplasmic EccB_5_ domain arrangement is crucial for the conformational dynamics and opening of the ESX-5 pore (see below).

### Structural basis of hexameric ESX-5 assembly and central pore

Next, we examined our structural model to identify key interactions that support hexameric ESX-5 assembly. Within the TM segment, one of the main interactions required for hexamerization occurs between the TM domains of EccB_5_ and EccC_5_. We observed that the EccC_5_ TMH1 of each protomer interacts with the EccB_5_ TMH of the neighboring protomer via hydrophobic interactions and is further stabilized by the EccC_5_ TMH2 (Fig. 2C). This domain swapping interaction was repeated in an anticlockwise fashion when viewed from the periplasm (Fig. 2D), thus generating an interlocking mechanism of next neighbor protomers within the TM section of the overall ESX-5 complex. The N terminus of EccB_5_, which hooks into the loop between EccD_5_-1 TMH10 and TMH11 of the neighboring protomer, mediates an additional interprotomer interaction at the cytoplasmic side (fig. S4F). Our model suggests that these EccB_5_-EccD_5_-1 interactions drive EccC_5_ TMH1 domain swapping within the TM section of the ESX-5 complex. In turn, this may lead to changes in the orientation of the EccB_5_ periplasmic domains, causing them to interlock and form a hexameric assembly in the periplasm. We propose that these steps are crucial in forming a secretion-competent complex with a central pore, required for substrate translocation.

**Fig. 3:**
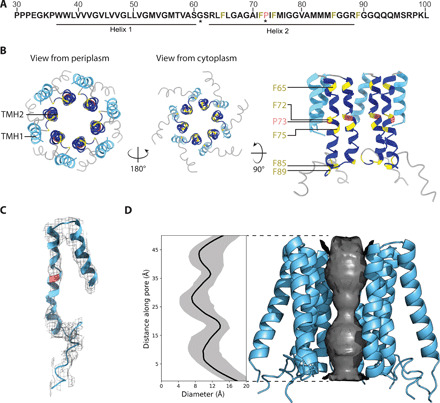
Central pore of the ESX-5 secretion system. (**A**) *M. xenopi* EccC_5_ sequence near the N terminus (residues 30 to 100) highlighting the two TMHs observed in the structure of the overall ESX-5 complex. Invariant residues are indicated by asterisk (*) (for further details see fig. S8). Colors correspond to the model shown in (B). (**B**) Top-scoring Rosetta (*36*) model of the central pore formed by EccC_5_ TMH1 (light blue) and TMH2 (dark blue) viewed from the periplasm, the cytoplasm, and along the membrane. Conserved residues have been colored (proline, red; phenylalanine, yellow). (**C**) EM density corresponding to the EccC_5_ TMHs, the top-scoring Rosetta model is shown. (**D**) Analysis of the pore diameter from 100 Rosetta models with HOLE (*42*), median pore diameter (black) and 90% confidence interval (gray). Side view of EccC_5_ pore helices of a top-scoring model showing the pore diameter analysis.

A notable feature of our ESX-5 core complex is the central pore (Fig. 3). The TMHs of EccC_5_ from each of the six protomers, most notably the TMH2 of one EccC_5_ subunit that forms a domain swapping interaction with TMH1 of the neighboring subunit, contribute to the formation of this central pore (Fig. 3). In contrast to most other TMHs of the complex, the density of TMH2 is less well defined (Fig. 3C), which implies that this helix is more flexible and adopts a range of orientations, as indicated by our calculated ensemble model (Fig. 3D). A highly conserved proline P73 (Fig. 3, A to C, and fig. S8) triggers a notable kink in the middle of TMH2, which results in increased flexibility of its C-terminal part. As observed in other transport proteins, proline residues positioned in related positions are key for their regulation and function (*12*, *13*),. Therefore, our structural findings suggest that P73 acts as a hinge point facilitating conformational flexibility of the pore required for substrate transport.

TMH2 also comprises a number of phenylalanine and methionine residues that are common to this helix across different ESX systems (Fig. 3A and fig. S8). In the ESX-5 structure, F66, F72, and F75 were observed to flank P73, orienting some of these aromatic side chains toward the inner surface of the pore and thus determining the pore diameter. To quantify this, we analyzed the diameter of the 100 highest-scoring ensemble models to sample the range of potential conformations of TMH2 (Fig. 3D). Our results showed that the narrowest constrictions of the central pore reduce the pore diameter to slightly below 1 nm. As this pore diameter is too narrow to secrete folded substrates with an estimated width of around 2.2 nm (*14*), we hypothesize that our model, in the absence of a bound substrate, represents a gated, secretion-competent state. Our structural findings indicate that even subtle changes in the interactions between the two EccC_5_ TMHs, the EccB_5_ TMH, and the innermost EccD_5_ TMH could induce conformational changes of the central ESX-5 pore, either toward a closed state or a more open state, to regulate substrate translocation. Comparing EccB angles between the hexameric ESX-5 pore complex and previously published dimeric ESX-3 subcomplexes (*9*, *10*) demonstrate inherent flexibility within this arrangement, supporting our hypothesis (fig. S7, C and D).

Analysis of the central pore of the ESX-5 system shows that this T7SS system displays intriguing similarities to other transport systems. The lining of secretion pores with bulky hydrophobic amino acids has been reported for other secretion machineries, in which the hydrophobic residues act to gate the pore, thus regulating secretion. For example, in A/B type toxins, such as the anthrax toxin from *Bacillus anthracis*, the phenylalanine rings or “Φ clamp” at the top of the channel restrict the passage of a folded lethal factor (*15*, *16*). In another example, a highly conserved Met-Met-Met loop of the export apparatus complex of T3SS forms a molecular gasket, which constricts the channel to less than 10 Å to preclude secretion (*17*). These systems do not share any sequence similarity with the ESX systems, suggesting that a highly dynamic state of the pore is a fundamental principle of bacterial secretion machineries that evolved through convergent mechanisms of different TM protein scaffolds.

## DISCUSSION

In conclusion, our cryo-EM structure of the hexameric *M. xenopi* ESX-5 pore complex reveals the architecture of the T7SS and the dynamic nature of the central secretion pore. According to our data, the considerably more flexible arrangements of the distal cytosolic ATPase domains of EccC_5_ and the dimer-of-trimers formed by the EccB_5_ periplasmic domains define the overall dynamics of the system, suggesting that major conformational changes occur during substrate recognition and substrate release into the periplasm.

**Fig. 4:**
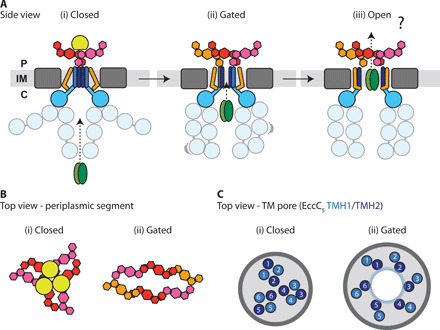
Scheme of conformational changes occurring in type VII secretion. (**A**) Side views of the ESX-5 overall conformations proceeding from an inactive to active complex. We propose that the closed state (i) based on the *M. tuberculosis* ESX-5 structure in the presence of the protease MycP_5_ (*18*) represents an inactive conformation. The second step toward active secretion (ii), based on the ESX-5 structure reported here, would represent a gated, secretion-competent conformation capable of initiating substrate translocation. In this conformation, the periplasmic EccB_5_ subunits are reorganized into a dimer-of-trimers arrangement, generating an elongated central cleft (**B**). The change in orientation of the EccB_5_ TMH reorients the EccC_5_ TMHs in the membrane to allow opening the central TM pore (**C**). In the closed conformation the same EccC_5_ TMHs are organized into four-helix bundles formed from two protomeric units without a visible pore. In all presently known structures, the cytosolic EccC_5_ domains adopt multiple conformations, demonstrating a high level of conformational plasticity requirements for ESX-5 substrate recognition and gating into the pore. We speculate that there could be an even more open conformation of the ESX-5 pore during active translocation of folded substrates, resulting from further reorientation of the EccB_5_ TMHs and the two pore forming EccC_5_ TMHs (iii). EccB_5_ is shown in orange, pink, and red; EccC_5_ is shown in blue and light blue; substrate is shown in brown; and MycP_5_ is shown in yellow. For reasons of clarity, the remaining structural EccD_5_ and EccE_5_ components of the TM section are shown in gray. In (C), the EccC_5_ TMH1 is in light blue and TMH2 is in dark blue. The TMHs have been numbered according to the protomer they correspond to. The numbering of the closed conformation is based on Bunduc *et al.* (*18*).

Following the submission of our work to a preprint server, another structure of a related ESX-5 complex from *Mycobacterium tuberculosis* was made available (*18*). In the structure, the presence of an additional conserved subunit with protease activity (MycP_5_) plugs the periplasmic EccB_5_ subunit domains in a triangular arrangement. In our experiments, MycP_5_, despite being present in the expression plasmid, does not stably associate with the purified *M. xenopi* ESX-5 complex, suggesting a regulatory rather than structural role of this subunit. Although in both structures there is an unexpected symmetry break between the TM-cytosolic region and the periplasmic domains, the nature of the EccB_5_ intersubunit interactions and the level of structural dynamics are distinct. These differences seem to correlate with distinct pore conformations in both structures: closed in the presence of MycP_5_ and more open in the absence of MycP_5_ (Fig. 4). Comparison of the two structural models suggests that the periplasmic arrangement of the EccB_5_ subunits and the level of conformational flexibility induced in the absence of MycP_5_ could play a role in the opening of the pore. As this hypothesis is based on the comparison of the presently available structural data only, functional data are still required for its ultimate validation. Whether there is further opening of the central pore to accommodate ESX-5 substrates destined for secretion, beyond the conformation observed in our structure (Fig. 4), remains to be established and will likely require structural information obtained in the presence of a bound substrate.

Among the diverse repertoire of bacterial secretion machineries characterized to date, the overall architecture of the T7SS is distinct. On the other hand, as the subunit composition in different T7SS is highly conserved, we argue that our findings on the overall architecture and associated conformational dynamics of the ESX-5 secretion machinery are generally applicable to other T7SS members. Our data provide a key step toward a comprehensive mechanistic understanding of distinct steps in T7SS-targeted translocation across the thick multilayered mycobacterial membrane. As tuberculosis (TB) remains as the longest lasting pandemic in the history of human civilization, and the recent rise of multidrug-resistant TB strains, development of novel anti-TB therapeutics remains a top societal priority. As ESX-5 represents a major determinant for mycobacterial pathogenicity, our structural findings may facilitate future target-driven drug discovery against TB and other mycobacterial diseases.

## MATERIALS AND METHODS

### Molecular biology

﻿Polymerase chain reaction (PCR) was performed using Q5 DNA polymerase (New England Biolabs). For cloning, *Escherichia coli* DH5α was used. The *eccD_5_* gene was amplified by PCR to include the N-terminal ubiquitin-like domain (residues 1 to 129) and inserted into the pMyNT vector using SliCE methods (*19*, *20*), generating pMyNT-EccD_5_^129^. The *M. xenopi* ESX-5 complex was expressed from pMV-ESX-5 vector in *M. smegmatis* as previously described (*8*).

### Protein expression and purification

Expression vectors were transformed into *M. smegmatis* mc^2^155 *groEL1*Δ*C* (*21*) and grown in Middlebrook 7H9 medium (BD Biosciences) supplemented with 0.2% (w/v) glucose (Carl Roth), 0.05% (v/v) Tween-80 (Carl Roth), and 0.2% (v/v) glycerol (Carl Roth) with appropriate antibiotics. For expression of the *M. xenopi* ESX-5 complex, cells were ﻿cultured to an optical density at 600 nm (OD_600_) of 1.5 and pelleted by centrifugation. EccD_5_^129^ was expressed using an inducible promoter, cells were grown to OD_600_ of 1.0, induced with 1% acetamide, cultured for further 24 hours at 37°C, and pelleted by centrifugation. The ESX-5 complex was purified as previously described in (*8*). The final sample was vitrified in 20 mM tris (pH 8) and 150 mM NaCl.

For the purification of EccD_5_^129^, cells were resuspended in buffer A [20 mM tris (pH 8.0), 300 mM NaCl, and 20 mM imidazole] with EDTA-free protease inhibitors (Roche) and deoxyribonuclease (Sigma-Aldrich). ﻿Cells were lysed by high-pressure emulsification, and unbroken cells were removed by centrifugation at 4°C for 20 min (19,000*g*).

### Cross-linking MS analysis

Fifty micrograms of purified ESX-5 complex was cross-linked by addition of an iso-stoichiometric mixture of H12/D12 isotope-coded, di-succinimidyl-suberate (DSS) (Creative Molecules). The cross-linking reaction (final concentration of 1 mM) was incubated for 30 min at 37°C and quenched by addition of ammonium bicarbonate to a final concentration of 50 mM for 10 min at 37°C. Cross-linked proteins were denatured using urea and RapiGest (Waters) [final concentration of 4 M and 0.05% (w/v)], respectively. Samples were reduced using 10 mM dithiothreitol (30 min at 37°C), and cysteines were carbamidomethylated with 15 mM iodoacetamide (30 min in the dark). Protein digestion was performed using 1:100 (w/w) LysC (Wako Chemicals) for 4 hours at 37°C and then finalized with 1:50 (w/w) trypsin (Promega) overnight at 37°C after the urea concentration was diluted to 1.5 M. Samples were then acidified with 10% (v/v) trifluoroacetic acid and desalted using OASIS HLB μElution Plate (Waters). Cross-linked peptides were enriched using size exclusion chromatography (SEC) (*22*).

Collected SEC fractions were analyzed by LC-coupled tandem MS (MS/MS) using a nanoACQUITY Ultra Performance LC (UPLC) system (Waters) connected online to linear ion trap quadrupole (LTQ)–Orbitrap Velos Pro instrument (Thermo Fisher Scientific). Peptides were separated on a BEH300 C18 (75 mm by 250 mm by 1.7 mm) nanoACQUITY UPLC column (Waters) using a stepwise 60-min gradient between 3 and 85% (v/v) acetonitrile in 0.1% (v/v) fusaric acid. Data acquisition was performed using a top-20 strategy, where survey MS scans (mass/charge ratio range of 375 to 1600) were acquired in the Orbitrap (*R* = 30,000), and up to 20 of the most abundant ions per full scan were fragmented by collision-induced dissociation (normalized collision energy = 40, activation *Q* = 0.250) and analyzed in the LTQ. To focus the acquisition on larger cross-linked peptides, charge state 1, charge state 2, and unknown were rejected. Dynamic exclusion was enabled with a repeat count = 1, exclusion duration = 60 s, list size = 500, and mass window of ±15 parts per million (ppm). Ion target values were 1,000,000 (or 500-ms maximum fill time) for full scans and 10,000 (or 50-ms maximum fill time) for MS/MS scans. The sample was analyzed in technical duplicates.

To assign the fragment ion spectra, raw files were converted to centroid mzXML format using a raw converter and then searched using xQuest (*23*) against a FASTA database containing the sequences of the cross-linked proteins. Posterior probabilities were calculated using xProphet (*23*), and results were filtered using the following parameters: false discovery rate = 0.05, minimum Δscore = 0.95, MS1 tolerance window of −4 to +7 ppm, and identity (Id) score > 36.

### X-ray crystallography and data processing

EccD_5_^129^ crystallized in initial conditions from the Morpheus screen (Molecular Dimensions) containing 0.06 M MgCl_2_, 0.03 M CaCl_2_, 0.1 M tris:bicine (pH 8.5), 10% OEG 20k, 20% poly(ethylene glycol) monomethyl ether 550. ﻿Diffraction data were collected at EMBL beamline P13 at the PETRA III storage ring (DESY, Hamburg, Germany). The data were processed with XDS (*24*) and merged with AIMLESS (*24*, *25*), and the relevant statistics are shown in table S1. We used the EccD_1_^129^ model from *M. tuberculosis* [Protein Data Bank (PDB) ID: 4KV2 (*26*)] as a molecular replacement candidate (45% sequence identity to *M. xenopi* EccD_5_). After the successful placement of the model using Phaser (*27*), manual building was performed in Coot (*28*). The model was refined using REFMAC5 (*29*).

### Cryo-EM sample preparation and data acquisition

For cryo-EM, 3.6 μl of the ESX-5 void peak fraction was applied on freshly glow-discharged Quantifoil R2/1 Cu 200 mesh grids with 2-nm continuous carbon. The sample was blotted for 2 s and vitrified in a liquid propane/ethane mix using a Vitrobot Mark IV at 10°C and 100% humidity. The grid was screened at the cryo-EM facility at the Centre for Structural Systems Biology (Hamburg, Germany), and high-resolution cryo-EM data were collected on a Titan Krios operated at 300 kV (Thermo Fisher Scientific FEI) equipped with a K3 direct detection camera (Gatan) and a BioQuantum K3 energy filter (Gatan) operated by SerialEM (*30*) at the EMBL Cryo-Electron Microscopy Service Platform (Heidelberg, Germany). A total of 27.873 movies with 40 frames were recorded in counting mode, with a total dose of 49.34 e/Å^2^ and a pixel size of 0.645 Å. The underfocus range was set to 0.7 to 1.7 μm, with a step size of 0.1 μm.

### Data processing

Data processing was performed in cryoSPARC (*31*) and is visualized in fig. S2. First, movie frames were aligned, and local motion was corrected for using patch-motion correction. The contrast transfer function (CTF) landscape of each micrograph was estimated using patch CTF estimation. The exposures were curated on the basis of local-motion distances and CTF-fit parameters. Particles were picked on the remaining 18,598 micrographs with a template-based particle picker. The templates were generated beforehand based on a map obtained from an initial dataset. The picked particles were inspected, and 635,219 particles were selected and subsequently extracted using a box size of 58 nm. For two-dimensional (2D) classification, the data were binned four times. Four rounds of 2D classification were performed. After each round, particles leading to intact classes were selected and included in the next round. A total of 284,402 particles passing these iterations were used to generate three ab-initio models. Particles corresponding to one class displaying a clear hexamer were further sorted with another round of 2D classification. The remaining selected 121,974 particles were reextracted and binned two times. An ab-initio model was generated, and the data were refined using the nonuniform refinement algorithm in the absence of any imposed symmetry (C1). The map and initial model building attempts revealed sixfold symmetry for the TM and membrane proximal-cytoplasmic regions. On the basis of this observation, we generated another map by imposing C6 symmetry. The resulting map at a global resolution of 3.4 Å revealed highest resolution in the TM regions (approximately 2.8 to 3.5 Å) and lowest resolution in the cytosolic part (approximately 4.0 to 5.5 Å).

To improve the resolution of the cytosolic part of each protomer, symmetry expansion was carried out followed by local refinement. The applied masks were created in UCSF Chimera (*32*) and processed in cryoSPARC (*31*). The generated map showed almost uniform distributed resolution for the cytoplasmic regions of about 3.0 Å. As no particle subtraction was performed beforehand, TMHs of the protomer are still visible in the map.

Heterogenous refinement with three classes was further performed on the 121,974 particles to investigate for different conformations of the periplasmic region. One class was chosen to assess the 3D variability (*31*) of cytoplasmic and periplasmic domains to further reduce heterogeneity. On the basis of the results, three clusters could be identified. One of them showed a keel conformation in the periplasmic region and was further refined with and without C2 symmetry. The two other clusters remained heterogenous likely due to the misalignment of different orientations. To improve the alignment, the low pass–filtered keel shape map (from cluster 1) was further refined and used as initial model for the refinement of the 121,974 particles. To achieve an improved separation of the keel shaped periplasmic domain, another round of heterogenous refinement was carried out with subsequent refinement of one class without imposed symmetry. The resulting map had a global resolution of 4.6 Å; however, the resolution of the periplasmic region is less than 10 Å.

### Atomic model building and refinement

As there are no reliable, high-resolution structures of any of the ESX-5 components or their homologs available in the PDB (*33*), we built de novo a model of the TM and nearby cytoplasmic regions of the complex (EccB_5_ 18-73; EccC_5_ 12-417; EccD_5_-1 23-502; EccD_5_-2 18-494; and EccE_5_ 95-332). An initial model was traced into a masked, focused refinement map using ARP/wARP cryo-EM module with default parameters (*34*). Next, domains for which we solved the high-resolution crystal structure (EccD_5_, residues 17 to 107; fig. S3, A to C) were fitted into the focused refinement map as rigid bodies using a Jiggle Fit tool from Coot (*28*) (fig. S6). The resulting model was completed manually using Coot in regions with local resolution allowing for unambiguous de novo model tracing. The interpretation of poorly resolved map regions was aided by alternative blurring and sharpening of the map in Coot. We used an iterative approach, where each manual model building step was followed with sequence assignment using findMySequence program (*35*), which allowed for an identification and correction of tracing errors (insertions and deletions). Loops that were resolved in the density but difficult to trace manually were built using the RosettaES density-guided enumerative-sampling algorithm from the Rosetta suite (*36*). The complete protomer model built into a focused refinement map was expanded to a complex using symmetry operations derived directly from the C6-symmetrized map using phenix.find_ncs_from_density (*37*) and completed manually in Coot. Apart from solving minor symmetry conflicts, we traced the model fragments that were resolved in the symmetrized map only. These included two TMHs of EccC_5_ (TMH1 and TMH2; residues 37 to 94). First, TMH1 was built de novo in Coot into the better resolved denisty and assigned to the sequence using findMySequence program (*35*), which allowed for an unambiguous determination of the helix direction and sequence register. Subsequently, the second TM helix (TMH2) was built using the RosettaES density-guided enumerative-sampling algorithm followed with refinement with C6 symmetry. We also added to the model the most distant to the central pore EccD_5_-2 helix (TMH11) based on a model of the corresponding helix in EccD_5_-1. Geometry of the models was improved in ChimeraX (*38*) using ISOLDE (*39*) tool. Last, models of the protomer and full complex were refined against corresponding maps using phenix.real_space_refine (*40*), with nonbonded restraints weight increased to 200. For the complex, additional restraints to the initial model coordinates and strict rotamer matching were used.

### Lipid analysis

To determine whether unassigned densities in the EM map may correspond to lipids copurified with the complex, we extracted lipids from purified ESX-5 samples using methanol-chloroform extraction. Cold methanol (200 μl) was added to 50 μg of ESX-5 complex in 20 mM tris (pH 8) and 150 mM NaCl and vortexed thoroughly. The sample was kept on ice, and 500 μl of cold chloroform and 200 μl of water were added and incubated for 10 min before centrifugation for 500 rpm for 5 min at 4°C. The phase-separated chloroform layer (300 μl) was removed and dried using a SpeedVac for 20 min at room temperature. Dried samples were resuspended in 100 μl of methanol before LC-MS analysis, performed as previously described (*41*).

### Pore dimension analysis

The local resolution of the central pore map region did not allow the determination side-chain conformations in the second EccC_5_ TM helix of (TMH2) and the reliable measurement of the pore dimensions. Therefore, we analyzed a whole ensemble of tentative models resulting from a density-guided enumerative sampling refinement with C6 symmetry restraints implemented in Rosetta. The pore profiles of 100 lowest-energy models were calculated using HOLE program (*42*).

### Integrative modeling

The models of the hexameric assembly of the periplasmic domains of EccB_5_ (amino acids 74 to 490) and cytoplasmic ATPase domains of EccC_5_ (amino acids 431 to 1390) were built using an integrative modeling protocol similar to what was previously used by us (*43*–*45*). The modeling procedure described in more detail below is implemented as a custom software based on Integrative Modeling Platform (*46*) version 2.13 and Python Modeling Interface (*47*) further described in (*48*). All additional code and input files necessary to reproduce the steps will be released on Zenodo repository upon publication.

The TM region of the ESX-5 structure built de novo as above and a homology model of the monomeric EccB_5_ periplasmic domain and EccC_5_ ATPase domains were used as input for modeling. The homology models of EccB_5_ and EccC_5_ were built using Modeller (*47*) based on the crystal structure of EccB_1_ of *M. tuberculosis* (PDB ID: 3X3M (*11*)) and EccC of *Thermomonospora curvata* (PDB ID: 4NH0 (*49*)) and using the sequence alignment obtained from the HHpred server (*50*). The nonsymmetrized (C1) EM map and available cross-links were used as modeling restraints. Owing to the low resolution (<10 Å) of the periplasmic and cytoplasmic regions, the high-frequency noise in the EM map was removed using a Gaussian filter with a SD of 3 Å for EccB_5_ and 5 Å for EccC_5_. In addition, to limit the conformational space, the fitting was performed using only a segment of the EM map not yet occupied by the TM region of the ESX-5 structure. The models were additionally restrained using high-confidence cross-links above an xQuest (*23*) ld score of 36. At this threshold, two and six cross-links could be mapped to the EccB_5_ and EccC_5_ sequences, respectively, and used for modeling (fig. S1D and table S4).

As the first step of the modeling, large libraries of alternative fits to the EM map of the monomeric EccB_5,_ and EccC_5_ structures were generated using the FitMap tool of the UCSF Chimera (*32*). The fitting was performed using 100,000 random initial placements, cross-correlation about the mean as the fitting score [Chimera’s “cam” score (*32*), equivalent to Pearson correlation coefficient], and the requirement of at least 80% of the input structure being covered by the EM map envelope defined at a permissive density threshold. This resulted in 9268 unique alternative fits for EccB_5_ and 5068 fits for EccC_5_ after clustering.

Second, the resulting alternative fits of the monomeric structures and the TM region of the ESX-5 structure were built de novo as above and used as input for the simultaneous fitting of six copies of EccB_5_ using the EM map and cross-link restraints and likewise for six copies of EccC_5_. The fitting was performed through simulated annealing Monte Carlo optimization that generates alternative configurations of the fits precalculated as above. The optimization was performed independently 4000 times with 12,000,000 Monte Carlo steps for each run for EccB_5_ and 2500 times with 12,000,000 Monte Carlo steps for each run for EccC_5_. The sampling exhaustiveness was assessed by ensuring that (i) the score converges in individual runs, (ii) no new better scoring models appear with extra runs, and (iii) the score distributions in two random samples of the models are statistically similar (fig. S6, A to C). The scoring function for the optimization was a sum of the EM fit restraint represented as the *P* values of the precalculated domain fits [calculated as described in (*43*–*45*)], cross-linking restraints, clash score, connectivity distance between neighboring domains, a term preventing overlap of the protein mass with the TM region, and a two- or sixfold symmetry restraint for EccB_5_ and EccC_5_, respectively. During the optimization, the structures were simultaneously represented at two resolutions—in Cα-atom representation and a coarse-grained representation—in which each 10-residue stretch was converted into one bead. The 10-residue bead representation was used for all restraints to increase computational efficiency except for the domain connectivity and cross-link restraints, for which the Cα-only representation was used for reasons of accuracy.

Last, top-scoring models from the previous step were subjected to a refinement coupled to an analysis of exhaustiveness of conformational sampling and estimation of model precision using a procedure proposed by Viswanath *et al.* (*51*). To this end, the models from the first modeling stage (simultaneous fitting based on the alternative fits) were split into two random subsets. The top 30 models from each subset were refined using a Monte Carlo simulated annealing optimization in which the structures were moved in the EM map with small rotational and translational increments. The scoring function consisted of cross-correlation to the EM map, domain connectivity restraint, clash score, a term preventing overlap of the protein mass with the TM region, and a two- or sixfold symmetry restraint as above. For EccC_5_, the monomeric homology model was split into two rigid bodies at the boundary between the ATPase domains 1 and 2, and an elastic network restraint was applied to enable limited flexibility between these domains. Each of the 30 models was refined with 200 independent runs with 260,000 steps. The top-scoring models from each of the two runs were selected, leading to two independent samples of refined models (about 1000 models in each sample). For EccC_5_, the multiple fitting step converged to a single top-scoring model; thus, only a single model was selected for the refinement, and the samples were generated by splitting the resulting refined models. The scores of the two samples were compared to each other to ensure convergence (fig. S5, D and E). The highest sampling precision at which sampling was exhaustive was determined on the basis of the root mean square deviation (RMSD) comparisons between all models and clustering at incremental RMSD thresholds using the statistical tests provided by Viswanath *et al.* (*51*) (fig. S5F). The two samples were then clustered at the resulting precision level (fig. S6G), and for each cluster, the model precision, defined as the average RMSD distance to cluster centroid, was calculated. The top 10 scoring models from all refined models were taken as the final ensemble model of the ESX-5 with the EccB_5_ (fig. S5H). All the top 10 models satisfied both EccB_5_ cross-link restraints (with a distance threshold of 30 Å; fig. S5I). The models will be deposited in the PDB-dev database upon publication.

## SUPPLEMENTARY MATERIALS

Supplementary material for this article is available at http://advances.sciencemag.org/cgi/content/full/7/26/eabg9923/DC1
